# Fermented Dairy Food Intake and Risk of Depression and Dementia in Later Life: Findings from a Prospective Cohort of Older Australians

**DOI:** 10.3390/nu18071020

**Published:** 2026-03-24

**Authors:** Muniratul Idrus, Dana Bliuc, Karen A. Mather, Henry Brodaty, Perminder S. Sachdev, Katya Numbers, Zhaoli Dai

**Affiliations:** 1 Centre for Big Data Research in Health, Faculty of Medicine and Health, University of New South Wales, Sydney 2033, Australia; munirahusna@gmail.com; 2 School of Population Health, Faculty of Medicine and Health, University of New South Wales, Sydney 2033, Australia; d.bliuc@bangor.ac.uk; 3 Centre for Healthy Brain Ageing (CHeBA), Discipline of Psychiatry and Mental Health, School of Clinical Medicine, Faculty of Medicine and Health, University of New South Wales, Sydney 2033, Australia; karen.mather@unsw.edu.au (K.A.M.); h.brodaty@unsw.edu.au (H.B.); p.sachdev@unsw.edu.au (P.S.S.); k.numbers@unsw.edu.au (K.N.); 4 School of Pharmacy, Faculty of Medicine and Health, University of Sydney, Sydney 2033, Australia; 5 Ageing Futures Institute, Faculty of Medicine and Health, University of New South Wales, Sydney 2033, Australia

**Keywords:** fermented food, dairy, yogurt, cheese, depression, dementia

## Abstract

**Background:** Fermented dairy foods, such as yogurt and cheese, contain bioactive components that differ from those in non-dairy foods, but their associations with depression and dementia risk in later life remain unclear. **Methods:** We analyzed data from the Sydney Memory and Ageing Study, a community-dwelling cohort of adults aged 70–90 years, to examine associations between dairy intake and depressive symptoms (Geriatric Depression Scale-15), psychological distress (Kessler-10), and incident depression (physician diagnosis or antidepressant use) and dementia (DSM-IV criteria). Intake of yogurt, cheese, and non-fermented milk was assessed at baseline using a validated food-frequency questionnaire. Longitudinal associations were examined using Fine–Gray competing-risks models that accounted for death; cross-sectional associations were also assessed. **Results:** Among 966 participants (mean age: 78.3; 55.5% women), compared with no consumption, higher yogurt intake (one standard serving) was significantly associated with lower depressive symptom scores (adjusted β: −0.37 and −0.39 for quartiles 3–4 (mean: 88.5–164 g/day), and so was low-fat cheese intake (mean: 13.2 g/day) (adjusted β: −0.35). Over a mean follow-up of 3.3 years, 120 incident cases of depression and 68 deaths occurred: higher yogurt intake and low-fat cheese consumption (versus non-consumption) were associated with lower risk of depression (adjusted subdistribution hazard ratios 0.41 [95% CI 0.19–0.88] and 0.40 [0.21–0.78], respectively). No significant associations were observed for psychological distress, cognition, or incident dementia (a mean follow-up of 5.2 years, 100 incident cases, and 153 deaths); no associations were observed for regular cheese or milk intake. **Conclusions:** These findings suggest a potential role for fermented dairy foods, particularly yogurt and low-fat cheese intake, but not non-fermented milk, in mental well-being in later life.

## 1. Introduction

Depression and dementia are major contributors to the global disease burden, particularly among older adults who face vulnerability from high rates of physical comorbidities, social isolation, and cognitive decline [1,2,3]. Globally, depression affects about 5.7% of adults over 60 [1], while dementia impacts an estimated 55 million people [1,4]. In Australia, depressive symptoms are present in roughly 10% of older adults, and dementia affects around 8–10% of people aged 65 and over [5,6]. As of 2025, dementia has become the leading cause of death among all Australians, regardless of age or sex [7]. This escalating toll has spurred a sharp rise in government mental health spending, which rose from $11.8 billion in 2018–19 to $13.2 billion in 2022–23 [8].

Given the detrimental impacts of dementia and depression on quality of life and healthcare expenditure, non-pharmacological approaches like dietary changes are garnering interest. Recent studies have suggested that increasing the intake of probiotic products may serve as a potential dietary intervention to prevent the onset and delay the progression of these conditions [9,10]. Fermented dairy, brimming with gut-friendly microbes [11] such as *Lactobacillus* and *Bifidobacterium* species [12,13], may offer therapeutic benefits by reducing neuroinflammation and supporting nervous system regulation, potentially improving neuropsychological health.

However, dairy foods exhibit heterogeneity in nutrient composition and biological activity. Fermented dairy products differ significantly from their non-fermented counterparts in terms of microbial loads, bioactive peptides, and lipid profiles. Despite this, most epidemiological studies lump total dairy intake together, potentially obscuring subtype-specific associations [14,15]. Moreover, longitudinal data on fermented dairy’s role in reducing depressive symptoms and dementia risk in older adults remains sparse [16,17]. Prior studies have often overlooked key competing risks like death, a critical blind spot in aging cohorts [18].

Hence, this study aimed to examine whether higher intake of fermented dairy products, particularly those lower in saturated fat, is associated with a reduced risk of incident depression and dementia, as well as its relationship with psychological distress, depressive symptoms, and cognitive function in a cohort of older Australians aged 70 or over, accounting for competing mortality risk in the longitudinal analysis. We hypothesized that such intake would be linked to lower risk of depression and dementia and better psychological and cognitive outcomes in older adults.

## 2. Methods

This study utilized data from the Sydney Memory and Ageing Study, a community-based longitudinal cohort established to examine cognitive and aging-related outcomes in older adults. Between 2005 and 2007, the study enrolled 1037 individuals aged 70–90 years from the Eastern suburbs of Sydney, Australia, all living independently and free of a dementia diagnosis at enrolment. Participants completed face-to-face assessments every 2 years for each follow-up wave, including comprehensive neuropsychological testing, a brief medical exam, and questionnaires on sociodemographic factors, health conditions, and lifestyle behaviors. Approximately 95% of participants had an informant who reported on the participant’s memory, thinking, and daily functioning. Informants were nominated by the participants and required to have at least 1 h of contact per week. Due to data availability, we report on participants followed up from baseline through Wave 3 (2009–2011) for incident depression and through Wave 4 (2011–2013) for incident dementia. Details of the cohort protocol have been published previously [19].

## 3. Ethics Approval

This study was approved by the University of New South Wales Human Research Ethics Committee (Approval Codes: HC 05037, 09382, 14327, 190962). The ethics approval was obtained on 22 February 2005, and all participants and their informants provided written consent to participate in the study.

## 4. Habitual Dietary Intake

At baseline, participants’ overall dietary intake over the past 12 months was measured using the validated Dietary Questionnaire for Epidemiological Studies version 2 (DQES v2), a food frequency questionnaire developed by the Cancer Council Victoria [20]. DQES v2 includes a food list of 74 items and six types of alcoholic beverages, grouped into cereal foods, sweets, snacks, dairy products, meats, fish, fruits, and vegetables.

## 5. Exposure of Interest

For dairy products, yogurt intake (regardless of fat content), regular cheese (hard, firm, or soft), and low-fat cheese (cottage or ricotta) were estimated in grams per day using the DQES v2 food frequency questionnaire. For each dairy type, we used “no intake” as the reference group. Milk intake (regular milk, including full-cream and reduced-fat milk) was analyzed for comparison with non-fermented dairy products. To ensure a sufficient distribution of incident cases across all intake levels within each dairy type, intake was classified using cohort-specific distributions: quartiles for yogurt and regular cheese, tertiles for milk, and binary categories for low-fat cheese.

## 6. Outcomes

### 6.1. Depressive Symptoms and Psychological Distress

Depressive symptoms were assessed using the 15-item Geriatric Depression Scale (GDS-15) [21]. Participants reported how they felt over the past week, with higher scores indicating more severe symptoms. Psychological distress was measured using the 10-item Kessler Psychological Distress Scale (K-10) [22].

### 6.2. Incidence of Depression

Incident depression was assessed among participants without depression at baseline, with follow-up extending through 2011 (Wave 3). Cases were ascertained using the following three criteria: self-reported physician diagnosis, current antidepressant use, or utilization of psychiatric treatments during the follow-up period. Depression onset was defined as the first time a participant fulfilled these criteria post-baseline.

### 6.3. Cognitive Decline Outcomes

Participants were assessed using a comprehensive battery of cognitive tests as described in the cohort profile [19]. Global cognition was calculated as the average of five composite cognitive domains: memory, processing speed, language, visuospatial function, and executive function [19]. Raw test scores for each domain were converted to composite scores and standardized as z-scores using the mean and standard deviation of the cognitively healthy baseline subsample. The higher the score (positive), the better the cognitive performance.

### 6.4. Incidence of Dementia

Incident dementia was assessed from baseline until six years later (Wave 4). Cases were adjudicated by a multidisciplinary consensus panel in neuropsychiatry, neuropsychology, and geriatric psychiatry, following the criteria of the *Diagnostic and Statistical Manual of Mental Disorders, fourth edition* (DSM-IV) [23]. Dementia onset was defined as the first time a participant fulfilled these criteria post-baseline.

## 7. Other Covariates

Baseline demographic information, dietary and lifestyle factors, and relevant health histories were included as covariates where relevant. These included age (years), sex (men, women), English-speaking background (English, non-English), level of education (no formal education, primary school, secondary school or higher), main occupation before retirement (managers/professionals, clerical/service, trade/labor, and homemakers or other); lifestyle factors such as body mass index (BMI) (kg/m^2^), cigarette smoking (never, former, current), alcohol use (never, former, current), total energy intake (kilojoules/day), dietary quality using a diet index (in quartiles) based on the food group recommendations of the Australian Dietary Guidelines [24], duration of moderate to vigorous physical activity (minutes/week), and social engagement estimated using the number of instances of in-person contact with someone per month. Relevant health histories included self-reported diabetes (yes, no), hypertension (yes, no), or antidepressant use; baseline GDS-15 score; and *APOE* ε4 carrier status (as previously described [19,25]).

## 8. Statistical Analysis

Among the eligible participants included in the analysis, their baseline characteristics were described across the quartiles of yogurt intake, including age, sex, language background, level of education, main occupation before retirement, BMI, cigarette smoking, alcohol use, total energy intake, dietary quality score, physical activity, social engagement, *APOE* ε4 carrier status, diabetes prevalence, hypertension prevalence, and baseline use of antidepressants. Missing data for covariates, including BMI, diet index, energy intake, and APOE ε4 carrier status, were minimal (<7% of the cohort). Our analysis included only participants with complete data.

### 8.1. Cross-Sectional Analysis

To examine the consistency of results for depression and dementia risks, we also assessed cross-sectional associations between dairy intakes and baseline measures of depressive symptoms (GDS-15), psychological distress (K-10), and global cognitive scores. Beta coefficients (β) with 95% confidence intervals (CIs) were estimated for each dairy food intake separately: yogurt and regular cheese (Q2–4 vs. Q1, no intake as reference), low-fat cheese (yes vs. no intake), and regular milk and reduced-fat milk (T2–3 vs. T1, no intake as reference) on all three outcomes.

### 8.2. Longitudinal Analysis

Eligible participants were prospectively followed up from their baseline interview (2005–2007) until the earliest of the following mutually exclusive events: incident depression or dementia diagnosis, death, voluntary withdrawal, loss to follow-up, or administrative censoring. Administrative censoring applied to event-free participants at the end of their respective follow-up periods—the final date of Wave 3 for incident depression and Wave 4 for incident dementia. Death from any cause was treated as a competing risk.

Associations between dairy intake and incident depression or incident dementia were estimated using Fine–Gray proportional subdistribution hazards models. The results are presented as subdistribution hazard ratios (sHRs) and 95% confidence intervals (CIs). This approach directly models the cumulative incidence function, providing estimates that more accurately reflect event probabilities in the presence of competing mortality, which is particularly relevant for aging populations. The proportional subdistribution hazards assumption was verified through log–log plots and time–exposure interaction tests, revealing no major violations. To evaluate the robustness of the primary findings and address potential reverse causality in the longitudinal analysis, sensitivity analyses were performed by excluding events occurring within the first 2 years of follow-up to minimize the influence of subclinical symptoms present at baseline.

### 8.3. Analysis of Non-Fermented Dairy Foods

In addition to fermented dairy products, we analyzed non-fermented dairy products (regular/reduced-fat milk) in relation to the aforementioned cross-sectional and longitudinal outcomes. This analysis contrasted fermented and non-fermented dairy products to explore whether the observed associations were specific to fermented dairy products rather than to total dairy intake.

All statistical analyses were conducted using Stata 18 (Stata Corporation 2023) for both univariable and multivariable models to assess consistency. All reported *p*-values were two-sided, and a value less than 0.05 was considered statistically significant.

## 9. Results

**Figure 1:**
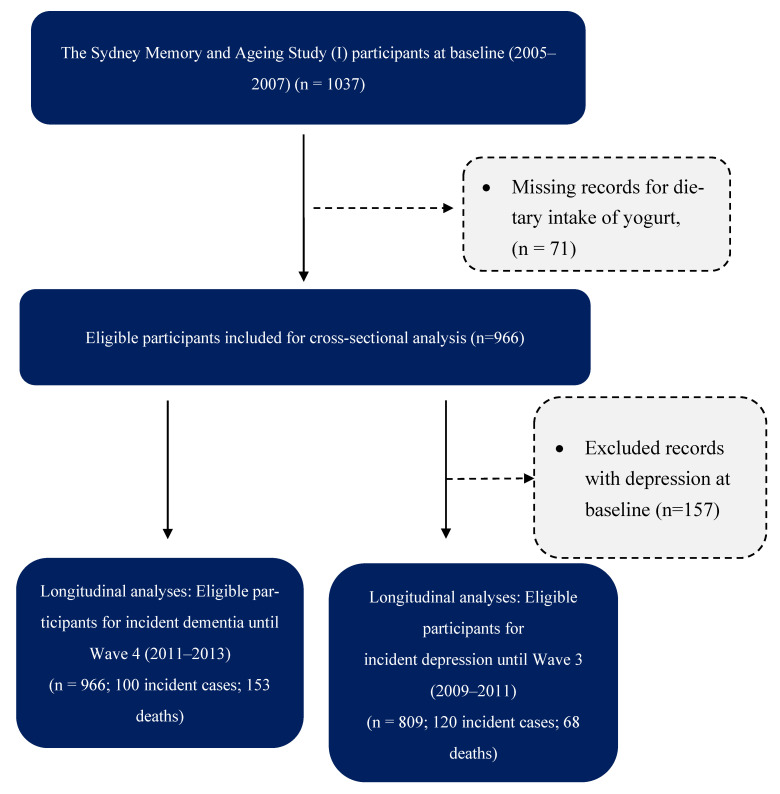
Participant selection for the cross-sectional and longitudinal analyses.

After excluding 71 participants with missing values for yogurt intake, 966 participants were eligible for a cross-sectional analysis of two depressive symptoms and cognitive function scores. As the cohort was dementia-free at baseline, 966 participants were eligible for the analysis of incident dementia after a mean follow-up of 5.2 years [Standard deviation (SD): 2.2 years], including 100 incident cases and 153 deaths. After excluding those with a history of depression at baseline (*n* = 157), 809 participants remained for analyses of incident depression after a mean follow-up of 3.3 (SD: 0.9) years, including 120 incident cases and 68 deaths. Figure 1 describes the process for the selection of participants for analysis.

Among 966 participants, yogurt intake increased markedly across quartiles, from 0 g/day in Q1 to 164.2 g/day in Q4, although the mean age was similar (77.6–78.9 years). Women were progressively more represented in higher-yogurt-intake groups, rising from 42.9% in Q1 to 66.5% in Q3, then reducing to 59.9% in Q4. Educational attainment was comparable across quartiles, with ~70% of individuals having a high school diploma or less. The main occupation before retirement was managerial or professional in the highest quartile of yogurt intake. BMI decreased modestly from 27.4 kg/m^2^ (Q1–Q2) to 26.6 kg/m^2^ in Q3. Lifestyle profiles also differed: those in Q1 engaged in the least physical activity (47.8 min/week) compared with those in Q4 (91.4 min/week). Total dietary quality scores increased across quartiles, from 40.6 in Q1 to 46.1 in Q4, indicating healthier overall diets among those with higher yogurt intake. Antidepressant use was over 10% and had the lowest usage rate of 11.0% in those with the highest yogurt intake. *APOE* ε4 carrier status, diabetes prevalence, and hypertension prevalence were relatively similar across groups. Total energy intake increased slightly among those with higher yogurt intake (6813 kJ/day in Q4 vs. 6267 kJ/day in Q1) (Table 1).

The regression analysis results were consistent across univariable and fully adjusted models. Therefore, all results presented here were from the fully adjusted models. In addition, no significant interaction between sex and yogurt intake was found, although women reported higher baseline yogurt intake than men. However, the sex-specific risk estimates suggest that higher yogurt intake was significantly associated with a lower risk of incident depression in women, but the associations for men alone were not statistically significant. However, the relationship using sex-specific risk estimates remained null for incident dementia (Supplementary Figure S4a,b).

### 9.1. Cross-Sectional Analysis

As illustrated in the Forest plot (Supplementary Figure S1), in the fully adjusted model, compared with non-consumers of yogurt (Q1), those in the third (Q3) or fourth quartile (Q4) had fewer depressive symptoms (Q3: adj. β: −0.37; 95% CI: −0.71, −0.02; and Q4: adj. β: −0.39; 95% CI: −0.77, −0.01). Similarly, consumption of low-fat cheese was associated with fewer depressive symptoms (adj. β: −0.35; 95% CI: −0.64, −0.05). No associations were found between regular cheese or milk intake and depression symptoms when assessed using GDS-15.

By contrast, no relationship was found between psychological distress, as estimated by the K-10 (Supplementary Figure S2), or global cognition scores (Supplementary Figure S3), and any of the assessed dairy food intakes.

### 9.2. Longitudinal Analysis

**Figure 2:**
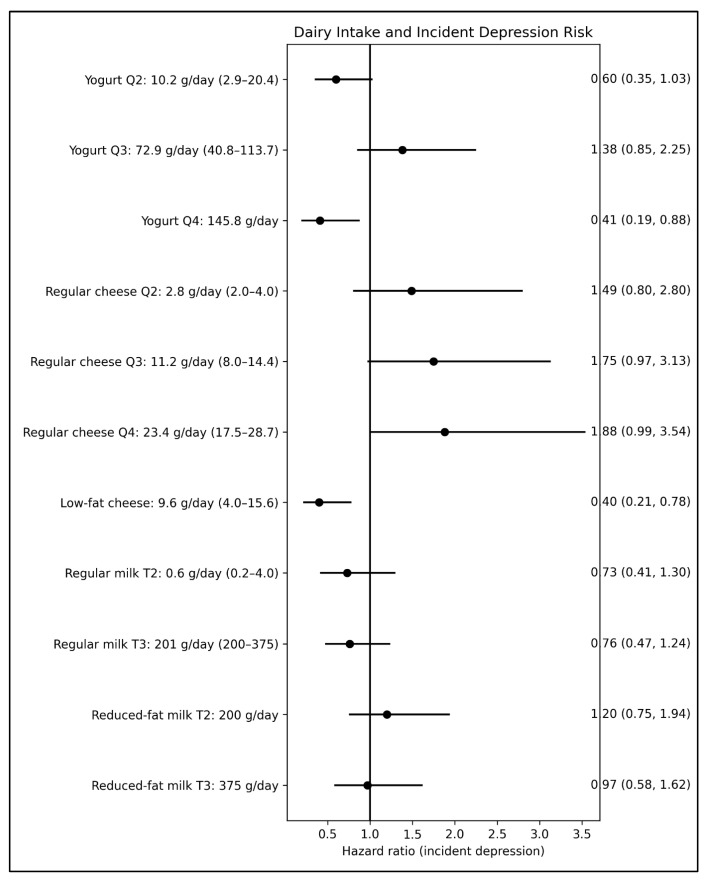
Forest plot for the relationships (subdistribution hazard ratios and 95% confidence interval) between dairy food intake at baseline and risk of incident depression (*n* = 120) over an average follow-up of 3.3 years (*n* = 809). All models used non-consumption of the respective dairy food as the reference group. Q: quartile; T: tertile. Values for food intake are reported as the median (interquartile range). Models were adjusted for age (years), sex (men, women), BMI (kg/m^2^), English-speaking background (English, non-English), level of education (no formal education, primary school, secondary school or higher), main occupation before retirement, cigarette smoking (never, former, current), alcohol use (never, former, current), total energy intake (kilojoules/day), dietary quality using a diet index (in quartile), duration of moderate to vigorous physical activity (minutes/week), social engagement (number of instances of in-person contact per month), self-reported diabetes (yes, no), hypertension (yes, no), geriatric depression scores (0–15), and competing risk of death.

**Figure 3:**
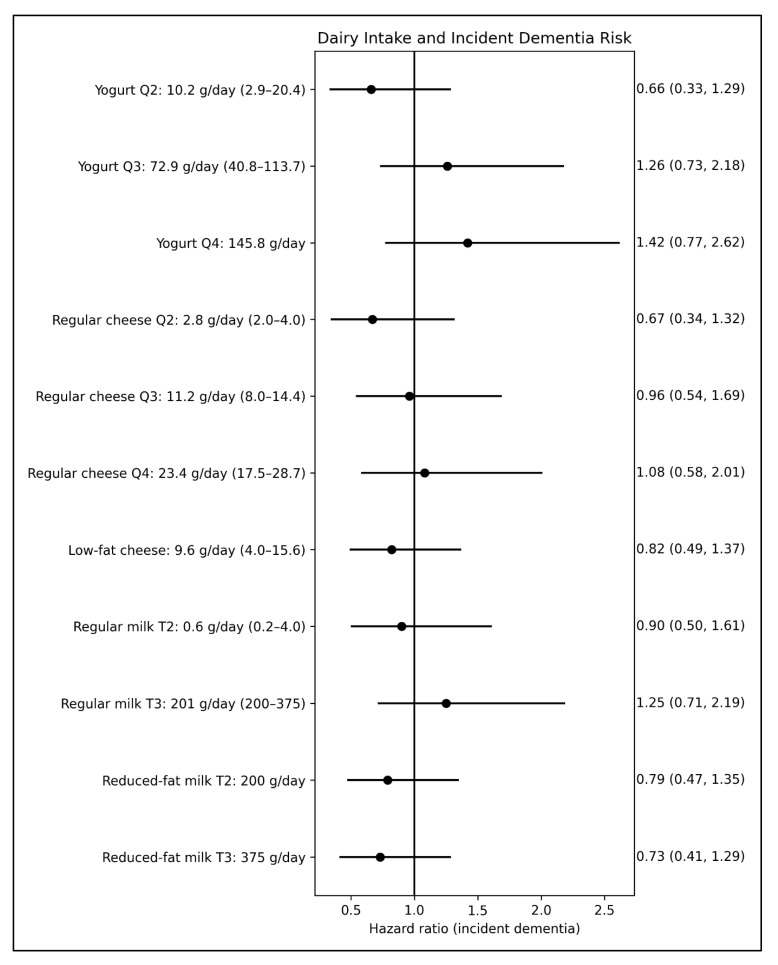
Forest plot for the relationships (subdistribution hazard ratios (sHRs) and 95% confidence interval) between dairy food intake and risk of incident dementia (*n* = 100) over an average follow-up of 5.2 years (N = 966). All models used non-consumption of the respective dairy food as the reference group. Q: quartile; T: tertile. Values for food intake are reported as the median (interquartile range). Models were adjusted for age (years), sex (men, women), English-speaking background (English, non-English), level of education (no formal education, primary school, secondary school or higher), BMI (kg/m^2^), main occupation before retirement, cigarette smoking (never, former, current), alcohol use (never, former, current), total energy intake (kilojoules/day), dietary quality using a diet index (in quartile), duration of moderate to vigorous physical activity (minutes/week), social engagement (number of instances of in-person contact per month), self-reported diabetes (yes, no), hypertension (yes, no), *APOE* ε4 carrier status (yes, no), and competing risk of death.

For incident depression (Figure 2), in the fully adjusted models, yogurt intake in the highest quartile was associated with a 59% reduction in risk compared with no intake (adj. sHR: 0.41; 95% CI: 0.19, 0.88). Similarly to the cross-sectional analyses for depressive symptoms, low-fat cheese intake was associated with a 60% lower risk of incident depression compared to no consumption (adj. sHR: 0.40; 95% CI: 0.21, 0.78). No association was found with either regular cheese intake or the intake of other dairy products. By contrast, none of the dairy intake variables, including yogurt and low-fat cheese, were associated with the risk of incident dementia over a mean follow-up of 5.2 years (Figure 3), suggesting that fermented dairy intake may not be relevant to cognitive decline or dementia risk in this cohort.

We further conducted sensitivity analyses by excluding 42 incident cases of depression that occurred within the first two years of follow-up since baseline. Our results were similar to those of the primary analysis: Comparing adults who had yogurt intake in quartile 4 with those in quartile 1, we found a lower risk of incident depression (sHR: 0.33; 95% CI: 0.12, 0.85; *p* = 0.022), and a similar result was obtained for low-fat cheese intake (consumption vs. no consumption: sHR: 0.51; 95% CI: 0.26, 1.03; *p* = 0.059); the results for other dairy intake categories were not statistically significant.

## 10. Discussion

The results from this prospective cohort of older Australians suggest that consumption of specific fermented dairy foods, particularly yogurt (approximately 1 standard serving per day) and low-fat cheese (approximately 1/2 slice per day), was associated with lower risk of depression-related outcomes and symptom burden in later life. Several prior studies have reported similar associations. In a cohort of Finnish men, higher intake of fermented dairy, particularly yogurt-like products, was associated with a 45% lower risk of depressive symptoms cross-sectionally and a lower risk of clinical depression longitudinally over 24 years (adj. HR: 0.55; 95% CI: 0.31, 0.99) [26].

In our study, we observed a stronger effect estimate for yogurt or cheese intake in association with a lower risk of depression. Although residual confounding remains a concern in observational studies, it is possible that our more pronounced effect estimates (59% reduction in yogurt; 60% reduction in low-fat cheese) may be partially attributable to healthy user bias. Since our cohort was predominantly white, well-educated, and urban-dwelling, we adjusted for occupation and education level in the models. Still, those who consumed more yogurt or low-fat cheese may engage in a broader cluster of health-seeking behaviors than those who consumed none of these dairy products. Nonetheless, our findings are similar to those of a meta-analysis of eight longitudinal cohort studies [17] which reported that fermented dairy intake overall was associated with a lower likelihood of depression in adults (OR: 0.89; 95% CI: 0.81, 0.98), with yogurt showing a stronger association than cheese (yogurt OR: 0.84; 95% CI: 0.72, 0.99; cheese OR: 0.91; 95% CI: 0.84, 0.98). Furthermore, consumption of yogurt or low-fat cheese may have amplified the protective associations by minimizing exposure to pro-inflammatory stimuli common in high-fat or high-sugar dairy products.

Notably, intake ranges in prior studies were similar to the amounts consumed observed in our cohort. NHANES data pertaining to yogurt reported lower odds of depression at moderate intake (approximately 1 serving/day), though this association was absent in the highest intake tier [27]. Such findings potentially support an association between moderate daily consumption of fermented dairy products and fewer depressive symptoms in later life. We speculate that fermented dairy products may improve mental health via the gut–brain axis. These foods contain Lactobacillus and Bifidobacterium, which may modulate gut microbiota composition, enhance short-chain fatty acid production, improve intestinal barrier integrity, and reduce systemic inflammation [28,29]. These pathways, therefore, could reduce lipopolysaccharide translocation, lower pro-inflammatory cytokine levels, and modulate neurotransmitter precursors and hypothalamic–pituitary–adrenal axis activity, collectively contributing to mood regulation [30,31].

The null associations observed for K-10 psychological distress may reflect its emphasis on anxiety and stress-related symptoms, which are more sensitive to acute psychosocial factors and less closely aligned with the mood-regulatory and anhedonic domains of depression that may be more responsive to dietary exposures [22,32].

While low-fat cheese intake was associated with fewer depressive symptoms and lower risk of incident depression, no such associations were observed for regular (full-fat) cheese. This divergence suggests that fat content may modify or counteract the potential mental health benefits of fermented cheese products. In this cohort, participants in the highest quartile of regular cheese intake consumed approximately 24.5 g/day, equivalent to roughly 30 g of cheese, providing an estimated 7–10 g of saturated fat, while low-fat cheese consumers averaged only 8 g/day with minimal saturated fat intake. Diets high in saturated fat may activate microglia, the brain’s resident immune cells, thereby triggering inflammatory cascades implicated in depressive symptoms [33], linked with increased reactive oxygen species and oxidative stress [34], as well as elevated pro-inflammatory cytokine levels [35]. These processes may contribute to the impairment of hippocampal neurogenesis, reduce synaptic plasticity, and promote neuroinflammation [36,37,38]. Taken together, the high saturated fat content of regular cheese could attenuate or negate the potential health benefits of fermented food, whereas low-fat fermented cheese may better preserve these benefits.

In contrast to the associations observed with depression outcomes, we found no relationship between fermented dairy intake and risk of global cognition or incident dementia risk. These null findings align with a previous systematic review reporting no significant associations between dairy intake and cognitive decline in middle-aged to older adults [15]. Although 5.2 years may appear short for a slow progressive condition such as dementia, a prior longitudinal analysis in the Sydney Memory and Ageing Study indicates that 9.5% of the cohort participants with no cognitive impairment developed dementia within six years [39], supporting the adequacy of the follow-up period in the current study. We recognize that the potential cognitive benefits of fermented dairy intake may be modest in magnitude and possibly related to specific domains, such as verbal memory or executive function, rather than global cognition [40]. However, such domain-specific analysis was beyond the scope of the current study, as the intention was to assess whether the longitudinal results for incident dementia were consistent with the baseline global cognition scores in the cross-sectional analysis.

The lack of association between non-fermented milk intake and any measured outcomes supports the specificity of the observed associations for fermented dairy. These findings suggest that fermentation-related properties, rather than dairy consumption per se, may be more relevant to late-life mental health outcomes. Overall, our findings add to the existing evidence by suggesting that, if present, associations are more likely to be limited to fermented dairy food than to milk.

This study has several strengths and limitations (Supplementary Table S1). First, we applied competing-risk models to account for death, a frequent and informative event in cohorts of adults aged 70 years and older, thereby providing more robust and clinically meaningful estimates of the associations between dairy intake and incident depression and dementia than conventional survival analyses. Failure to account for competing mortality may distort associations between dietary exposures and late-life neuropsychological outcomes. Second, the detailed dietary assessment enabled differentiation between fermented and non-fermented dairy products, as well as between low-fat and regular varieties, distinctions rarely examined in prior studies, allowing us to evaluate the potential modifying role of saturated fat content alongside fermentation status. Third, the cohort benefited from extensive neuropsychological testing, expert clinical diagnoses, and validated assessments of depressive symptoms and psychological distress, together with comprehensive data on medical history, physical activity, and social factors. This depth of phenotyping permitted careful adjustment for key confounders, strengthening the internal validity of the findings.

Several limitations should also be acknowledged. First, dietary intake was measured using a food frequency questionnaire (FFQ), which is subject to recall bias and could attenuate true associations through misclassification. Second, our FFQ did not capture detailed information on specific probiotic strains, added sugar levels, or lipid profiles of dairy products consumed, as such data are rarely amenable to accurate participant recall. Consequently, we were unable to differentiate how particular formulations or brands contributed to the observed neuropsychological outcomes. Third, we relied on a single baseline dietary assessment, which may not capture temporal changes in dietary intake. However, given that dietary patterns among adults aged 55 years and older are generally stable over at least four years [41], a baseline assessment is appropriate for our follow-up durations of 3.3 years for incident depression and 5.2 years for incident dementia. Fourth, despite adjustment for diet quality and key covariates, residual confounding from unmeasured dietary and biological factors cannot be completely ruled out. With respect to other fermented foods, the intake of kimchi, kombucha, and pickled vegetables is relatively uncommon in the general Australian population. As these foods were not captured in the administered FFQ, their contribution to dietary intake in this cohort is likely to be minimal. Regarding overall gut health, there was no widely accepted clinical or epidemiological measure that could be derived from the available dataset. While gut–brain interactions are a growing area of research interest, the role of overall gut health in neuropsychological outcomes remains incompletely understood. Therefore, we could not adjust for this factor in our models. Reverse causation remains a possibility, especially in cross-sectional analysis, as pre-existing depressive symptoms may have influenced dietary patterns and fermented dairy intake. The longitudinal analysis is appropriate in providing evidence of a potential association sequentially, as individuals with a history of depression or dementia were excluded at baseline, when diet intake was assessed. Finally, the generalizability of these findings may be limited to other socioeconomic, cultural, and ethnic groups, as the vast majority of participants were white, well-educated, and from English-speaking backgrounds, residing in urban Sydney.

## 11. Conclusions

This study of older Australians suggests that moderate intake of fermented dairy products, particularly yogurt and low-fat cheese, was associated with fewer depressive symptoms and a lower risk of incident depression, whereas no associations were observed for cognitive outcomes or dementia. These findings add to the growing evidence that fermented dairy products may be a simple, widely accessible dietary component with potential relevance to mental health in later life. Although the longitudinal design is a strength, these observational data do not establish a causal relationship. Larger prospective studies in diverse populations and well-designed randomized trials are warranted to confirm these findings and clarify whether specific fermented dairy products, intake levels, and consumption patterns can improve mental well-being in older adults.

## Figures and Tables

**Table 1:** Baseline characteristics of the study participants according to yogurt intake quartile levels (*n* = 966).

	Total	Quartile 1 (Lowest Quartile, No Intake)	Quartile 2	Quartile 3	Quartile 4 (Highest Quartile)
**N**	966 (100.0%)	303 (31.4%)	293 (30.3%)	188 (19.5%)	182 (18.8%)
**Yogurt intake (g/day), mean (SD)**	53.7 (67.8)	0	18.1 (15.0)	88.5 (20.0)	164.2 (57.4)
**Age (years)**	78.39 (4.8)	78.9 (5.0)	77.9 (4.8)	77.6 (4.4)	78.5 (4.9)
**Sex**					
Men	430 (44.5%)	173 (57.1%)	121 (41.3%)	63 (33.5%)	73 (40.1%)
Women	536 (55.5%)	130 (42.9%)	172 (58.7%)	125 (66.5%)	109 (59.9%)
**Education level**					
High school or below	679 (70.3%)	215 (71.0%)	203 (69.3%)	135 (71.8%)	126 (69.2%)
Vocational education and training	87 (9.0%)	25 (8.3%)	28 (9.6%)	17 (9.0%)	17 (9.3%)
University or above	200 (20.7%)	63 (20.8%)	62 (21.2%)	36 (19.1%)	39 (21.4%)
**English-speaking background**					
English	816 (84.5%)	265 (87.5%)	242 (82.6%)	156 (83.0%)	153 (84.1%)
Non-English	150 (15.5%)	38 (12.5%)	51 (17.4%)	32 (17.0%)	29 (15.9%)
**Main occupation before retirement**					
Manager/professional	481 (49.8%)	139 (45.9%)	154 (52.6%)	90 (47.9%)	98 (53.8%)
Clerical/service	335 (34.7%)	121 (39.9%)	85 (29.0%)	70 (37.2%)	59 (32.4%)
Trade/labor	94 (9.7%)	22 (7.3%)	41 (14.0%)	17 (9.0%)	14 (7.7%)
Homemakers or other	56 (5.8%)	21 (6.9%)	13 (4.4%)	11 (5.9%)	11 (6.0%)
**BMI (kg/m^2^)**	27.2 (4.5)	27.4 (4.5)	27.4 (4.7)	26.6 (4.1)	26.9 (4.6)
**Smoking status**					
Never smokers	447 (46.3%)	143 (47.2%)	139 (47.4%)	88 (46.8%)	77 (42.3%)
Former smokers	485 (50.3%)	147 (48.5%)	144 (49.1%)	97 (51.6%)	97 (53.6%)
Current smokers	34 (3.5%)	10 (3.3%)	15 (5.1%)	4 (2.1%)	5 (2.8%)
**Alcohol consumption**					
Never drinkers	52 (5.4%)	18 (5.9%)	16 (5.5%)	10 (5.3%)	8 (4.4%)
Former drinkers	68 (7.0%)	17 (5.6%)	22 (7.5%)	14 (7.4%)	15 (8.2%)
Current drinkers	841 (87.1%)	264 (87.1%)	257 (87.7%)	163 (86.7%)	157 (86.3%)
**Moderate or vigorous physical activity** (minutes/week)	74.4 (162.7)	47.8 (122.7)	84.8(175.3)	84.6 (189.2)	91.4 (166.4)
**Social engagement** (in-person contact, times/month)	2.2 (0.8)	2.25 (0.89)	2.22 (0.7)	2.20 (0.7)	2.20 (0.8)
**Total energy intake** (kilojoules/day)	6426.2 (2081.2)	6267.4 (2062.4)	6440.4 (2141.5)	6290.1 (2010.2)	6813.3 (2051.5)
**Dietary index score**	43.8 (10.1)	40.6 (10.2)	43.9 (9.9)	46.4 (9.3)	46.1 (9.7)
**Antidepressant use at baseline**					
No	842 (87.2%)	265 (87.5%)	255 (87.0%)	160 (85.1%)	162 (89.0%)
Yes	124 (12.8%)	38 (12.5%)	38 (13.0%)	28 (14.9%)	20 (11.0%)
***APOE *** **ε4 carrier**					
Non-carrier	717 (77.1%)	229 (77.9%)	222 (78.7%)	133 (75.1%)	133 (75.1%)
Carrier	213 (22.9%)	65 (22.1%)	60 (21.3%)	44 (24.9%)	44 (24.9%)
**Diabetes prevalence**					
No	586 (60.7%)	178 (58.7%)	178 (60.8%)	118 (62.8%)	112 (61.5%)
Yes	380 (39.3%)	125 (41.3%)	115 (39.2%)	70 (37.2%)	70 (38.5%)
**Hypertension prevalence**					
No	135 (14.0%)	39 (12.9%)	39 (13.3%)	26 (13.8%)	31 (17.0%)
Yes	831 (86.0%)	264 (87.1%)	254 (86.7%)	162 (86.2%)	151 (83.0%)

## Data Availability

The data presented in this study are available at the request of the corresponding author and the founders of the Sydney Memory Ageing Study. The data is not publicly available due to privacy or ethical restrictions.

## Supplementary Materials

The following supporting information can be downloaded at: https://www.mdpi.com/article/10.3390/nu18071020/s1, Figure S1: Forest plot for the relationships (beta coefficient and 95% confidence interval) between dairy food intake and depressive symptoms score measured by the 15-item Geriatric Depression Scale (GDS15) in cross-sectional analyses (n = 966). Figure S2. Forest plot for the relationship (beta coefficient and 95% confidence interval) between dairy food intake and psychological distress measured by Kessler-10 in cross-sectional analysis (n = 966). Figure S3: Forest plot for the relationships (beta coefficient and 95% confidence interval) between dairy food intake and cognitive function measured by standardized global cognition score in cross-sectional analysis (n = 966). Figure S4a: Forest plot for the sex-specific relationships (subdistribution hazard ratios (sHRs) and 95% confidence interval) between dairy food at baseline and incident depression (men: n = 85, women n = 35) over an average of 3.3 years follow-up (men: N = 370, women N = 439). Figure S4b. Forest plot for the sex-specific relationships (subdistribution hazard ratios (sHRs) and 95% confidence interval) between dairy food intake at baseline and incident depression (men: n = 51, women n = 49) over an average of 5.2 years follow-up (men: N = 430, women N = 536); Table S1: Methodological appraisal of the study design, exposure assessment, and statistical framework.
