# Supplementary material for: Fermented Dairy Food Intake and Risk of Depression and Dementia in Later Life: Findings from a Prospective Cohort of Older Australians
Source: Nutrients. 2026 Mar 24;18(7):1020. doi: 10.3390/nu18071020 (PMC13075031; doi:10.3390/nu18071020)
Supplement: Supplementary file 1 [file nutrients-18-01020-s001.zip › nutrients-4138906-supplementary.pdf]

Supplementary materials

Supplementary Table S1. Methodological appraisal of the study design, exposure assessment, and statistical framework.

Attribute	Detail
Strengths	
Statistical Analysis	Use of subdistribution hazard models (Fine-Gray) to account for the competing risk of mortality in an older population aged 70 years and older, reducing survival bias.
Exposure Characterization	Detailed dietary assessment included both fermented and non-fermented dairy foods, with low-fat/full-fat varieties for milk and cheese, allowing evaluation of fermentation status and saturated fat as potential effect modifiers.
Outcome Rigor	Extensive neuropsychological testing, expert-adjudicated clinical diagnoses, and validated assessments of depressive symptoms and psychological distress allowed rigorous clinical phenotyping.
Covariate Control	Comprehensive data on medical history, physical activity, and social factors facilitated adjustment for potential confounding.
Limitations	
Exposure Assessment	Reliance on a self-reported food frequency questionnaire, which is subject to recall bias and may lead to misclassification.
Granularity of Data	The absence of data on specific probiotic strains, sugar content, or fat composition limits the ability to assess the independent or synergistic effects of these components on mental health outcomes. However, this information is unlikely to be collected or recalled in an FFQ.
Causal Inference	The observational nature of the data precludes causal conclusions, and residual confounding from unmeasured factors (e.g., intake of other fermented foods and other underlying health conditions) remains possible.
Reverse Causation	Potential reverse causation remains a concern in cross-sectional analyses, as pre-existing depressive symptoms may have influenced dietary patterns and fermented dairy consumption.
Generalizability	The cohort was predominantly White, well-educated, and urban-dwelling, which may limit the generalizability of the findings to other socioeconomic groups or older adults from other cultural or ethnic backgrounds.

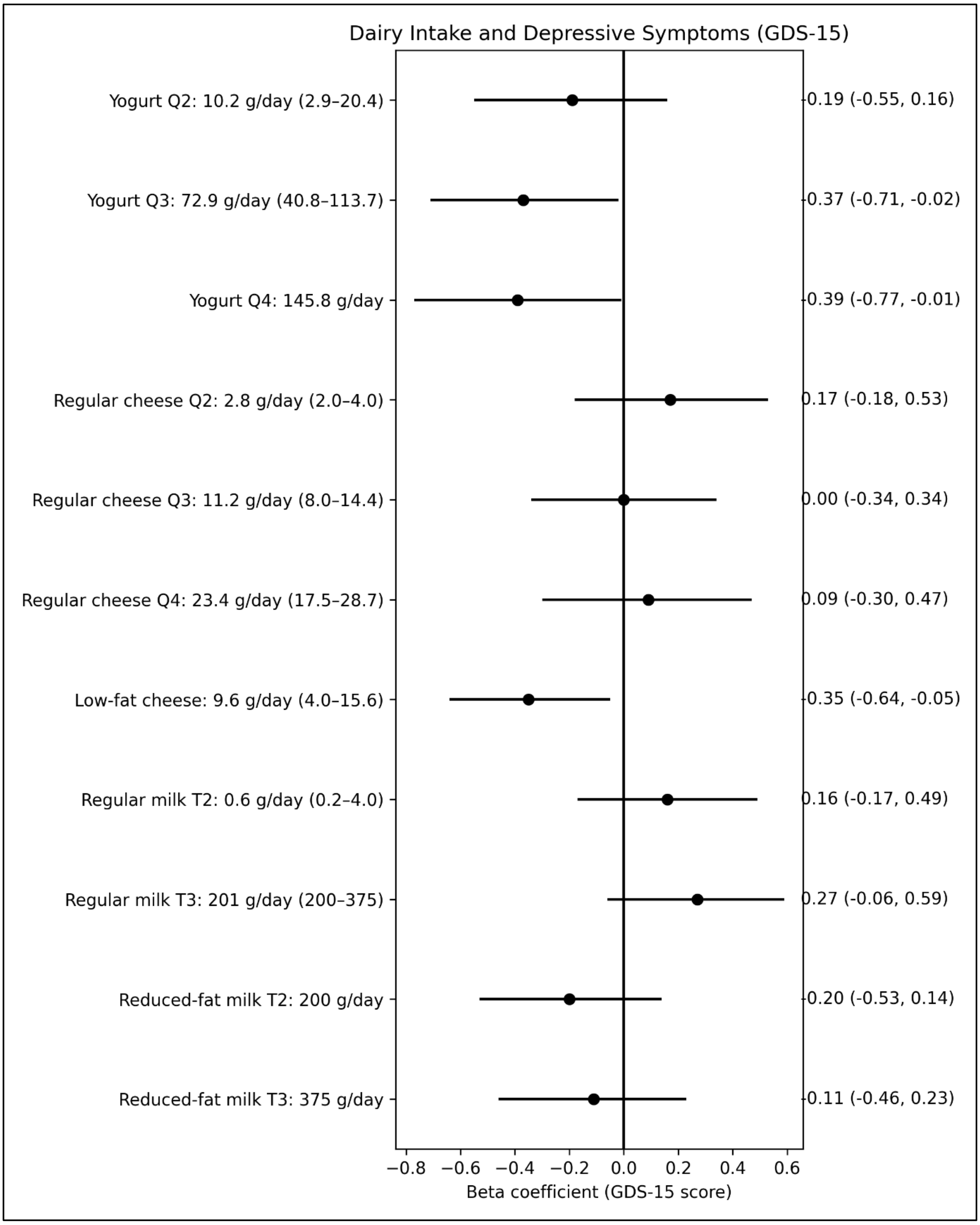

Supplementary Figure S1. Forest plot for the relationships (beta coefficient and 95% confidence interval) between dairy food intake and depressive symptoms score measured by the 15-item Geriatric Depression Scale (GDS15) in cross-sectional analyses (n=966). *All models used non-consumption of the respective dairy food as the reference group. Q: quartile; T: tertile. Values for food intake are reported as median (interquartile range). **Models were adjusted for age, sex, English-speaking background, level of education, main occupation before retirement, BMI (kg/m^2), cigarette smoking (never, former, current), alcohol use (never, former, current), total energy intake (kilojoules/day), dietary quality, duration of moderate to vigorous physical activity (minutes/week), and social engagement (in-person contacts per month), self-reported diabetes (yes, no), hypertension (yes, no), and antidepressant use at baseline (yes, no).

https://doi.org/10.3390/xxxxx

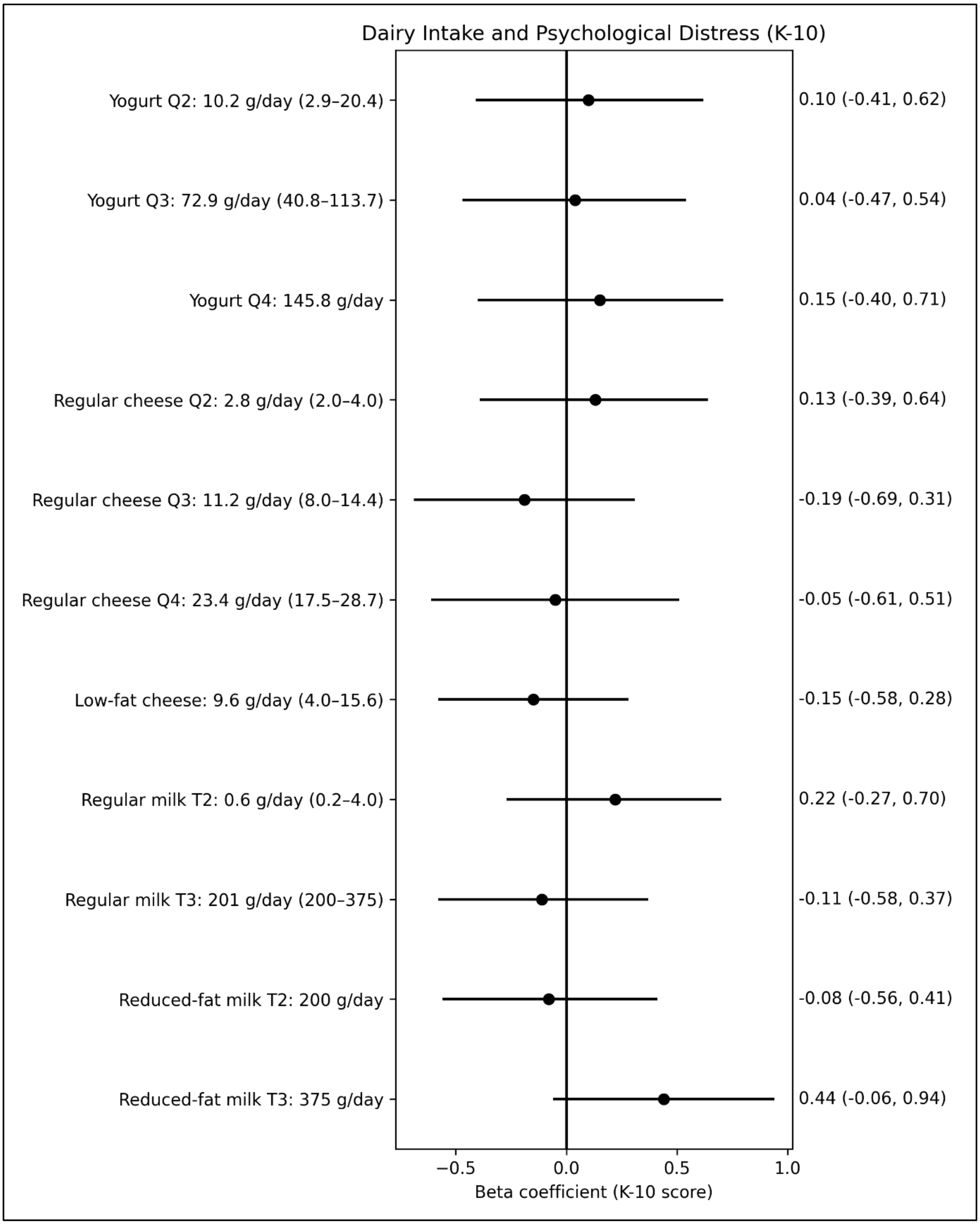

Supplementary Figure S2. Forest plot for the relationship (beta coefficient and 95% confidence interval) between dairy food intake and psychological distress measured by Kessler-10 in cross-sectional analysis (n=966). *All models used non-consumption of the respective dairy food as the reference group. Q: quartile; T: tertile. Values for food intake are reported as median (interquartile range). **Models were adjusted for age (years), sex (men, women), English-speaking background (English, non-English), level of education (no formal education, primary school, secondary school or higher), main occupation before retirement, BMI (kg/m^2), cigarette smoking (never, former, current), alcohol use (never, former, current), total energy intake (kilojoules/day), dietary quality, duration of moderate to vigorous physical activity (minutes/week), and social engagement (in-person contacts per month), self-reported diabetes (yes, no), hypertension (yes, no), and antidepressant use at baseline (yes, no).

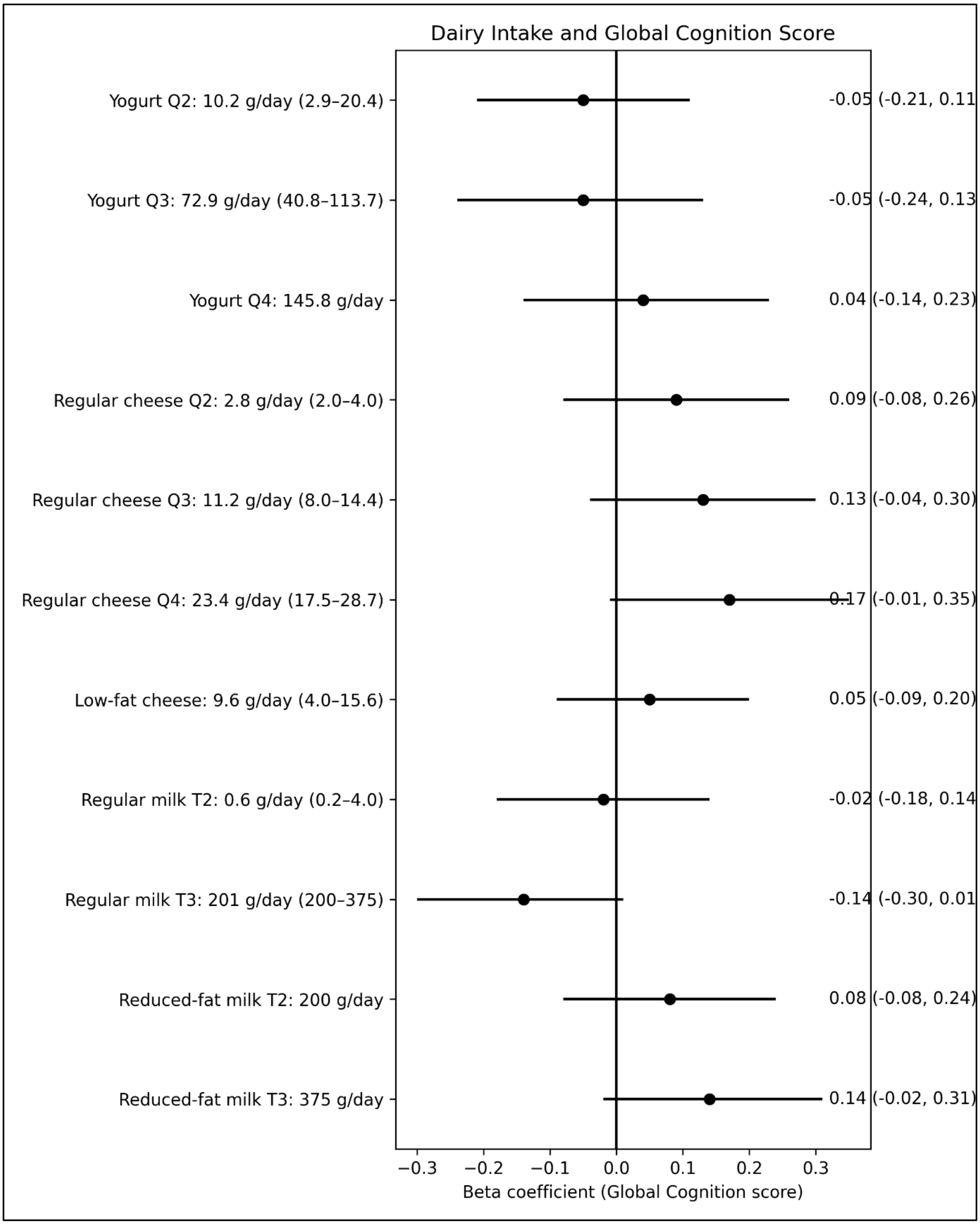

Supplementary Figure S3. Forest plot for the relationships (beta coefficient and 95% confidence interval) between dairy food intake and cognitive function measured by standardized global cognition score in cross-sectional analysis (n=966). All models used non-consumption of the respective dairy food as the reference group. Q: quartile; T: tertile. Values for food intake are reported as median (interquartile range). Models were adjusted for age (years), sex (men, women), English-speaking background (English, non-English), level of education (no formal education, primary school, secondary school or higher), main occupation before retirement, BMI (kg/m^2), cigarette smoking (never, former, current), alcohol use (never, former, current), total energy intake (kilojoules/day), dietary quality, duration of moderate to vigorous physical activity (minutes/week), and social engagement (in-person contacts per month), self-reported diabetes (yes, no), hypertension (yes, no), and *APOE* ε4 carrier status (yes, no).

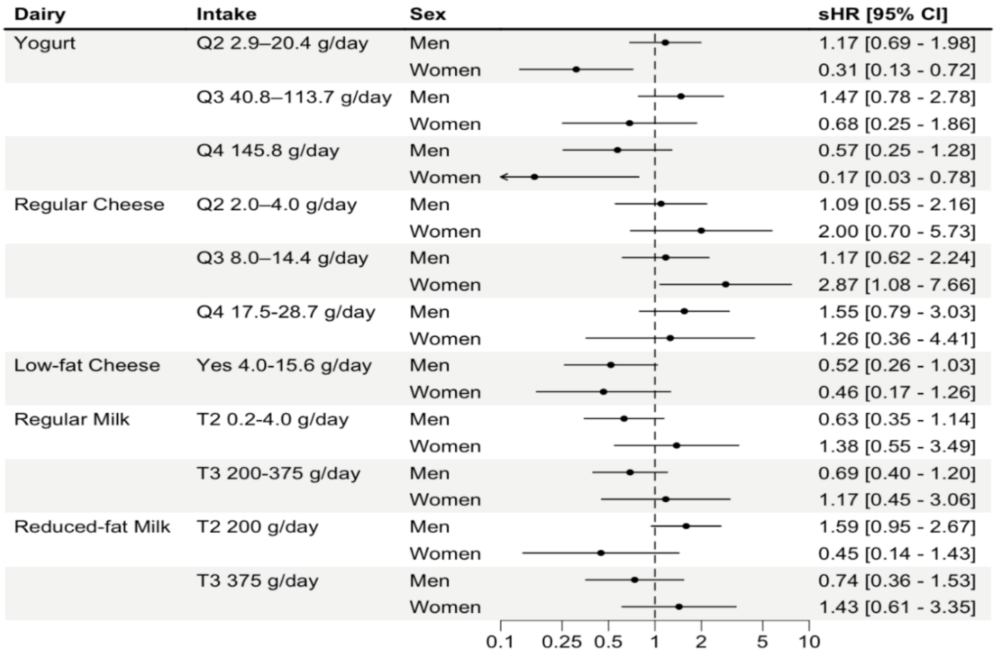

Supplementary Figure S4 a. Forest plot for the sex-specific relationships (subdistribution hazard ratios (sHRs) and 95% confidence interval) between dairy food at baseline and incident depression (men: n=85, women n=35) over an average of 3.3 years follow-up (men: N=370, women N=439). All models used non-consumption of the respective dairy food as the reference group. Q: quartile; T: tertile. Values for food intake are reported as median (interquartile range). Models were adjusted for age (years), English-speaking background (English, non-English), level of education (no formal education, primary school, secondary school or higher), BMI (kg/m2), main occupation before retirement, cigarette smoking (never, former, current), alcohol use (never, former, current), total energy intake (kilojoules/day), dietary quality using a diet index (in quartile), duration of moderate to vigorous physical activity (minutes/week), and social engagement (in-person contacts per month), self-reported diabetes (yes, no), hypertension (yes, no), APOE ε4 carrier status (yes, no), and competing risk of death.

https://doi.org/10.3390/xxxxx

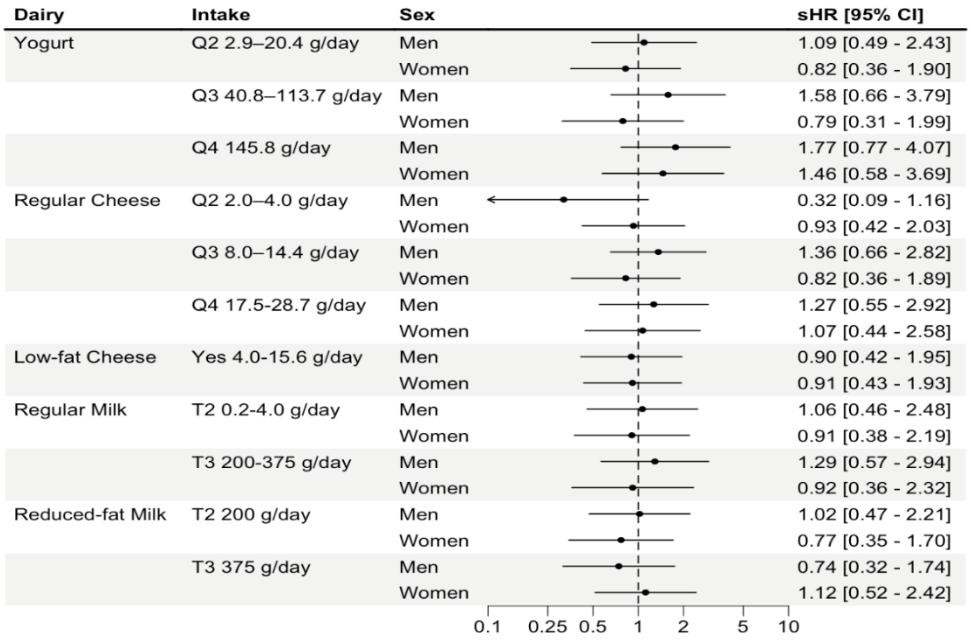

Supplementary Figure S4 b. Forest plot for the sex-specific relationships (subdistribution hazard ratios (sHRs) and 95% confidence interval) between dairy food intake at baseline and incident depression (men: n=51, women n=49) over an average of 5.2 years follow-up (men: N=430, women N=536). All models used non-consumption of the respective dairy food as the reference group. Q: quartile; T: tertile. Values for food intake are reported as median (interquartile range). Models were adjusted for age (years), sex (men, women), English-speaking background (English, non-English), level of education (no formal education, primary school, secondary school or higher), BMI (kg/m2), main occupation before retirement, cigarette smoking (never, former, current), alcohol use (never, former, current), total energy intake (kilojoules/day), dietary quality using a diet index (in quartile), duration of moderate to vigorous physical activity (minutes/week), and social engagement (in-person contacts per month), self-reported diabetes (yes, no), hypertension (yes, no), APOE ε4 carrier status (yes, no), and competing risk of death.

https://doi.org/10.3390/xxxxx
